# Body Dysmorphic Disorder Insights in an Inpatient Psychiatric Setting

**DOI:** 10.1155/2021/6636124

**Published:** 2021-05-19

**Authors:** Zachary A. Koenig, Sarah Callaham, Brittany Waltz, Julie Bosley, Raja Mogallapu, Michael Ang-Rabanes

**Affiliations:** ^1^West Virginia University School of Medicine, USA; ^2^West Liberty University, USA; ^3^Department of Behavioral Medicine and Psychiatry, West Virginia University, USA

## Abstract

Body dysmorphic disorder is a chronic disorder involving imagined or partial appearance defects that lead to significant impairment in everyday life. It is quite prevalent but remains a clinically underdiagnosed psychiatric condition especially in the inpatient psychiatric setting. Onset of body dysmorphic disorder typically begins in adolescence with subclinical symptoms. Over time, symptoms progress to patients meeting the full *Diagnostic and Statistical Manual of Mental Disorders*, 5th edition (DSM-5) criteria. Severe cases of the body dysmorphic disorder are often camouflaged by concurrent diseases like major depressive disorder, obsessive-compulsive disorder, substance use disorder, and social anxiety disorder. Further, compounding the complexity of body dysmorphic disorder is a treatment of patients who present with coinciding suicidal ideations. Here, we present a unique case of a 40-year-old female admitted to an inpatient psychiatric unit for treatment of ongoing depression and suicidal symptoms. Early on in her inpatient course, she had symptoms of obsessive-compulsive disorder, social anxiety disorder, and alcohol use disorder. The constellation of symptoms prompted evaluation for body dysmorphic disorder and subsequent targeted treatment. This case report highlights the complexities associated with diagnosing body dysmorphic disorder, the importance of considering it a branch point for other psychiatric conditions, and the treatment for patients who present with coinciding suicidal behavior.

## 1. Introduction

Body dysmorphic disorder (BDD) is a psychiatric condition in which a patient is concerned about their physical features such that they feel deformed, repulsive, or unattractive when in fact this is not an accurate perception (Table 1). These preoccupations with physical appearance lead to profound distress and impairment in both social and occupational aspects of life [1]. Patients often perform repetitive actions including mirror checking, excessive grooming, comparing themselves to others, skin picking, and compulsive shopping to mitigate these perceived abnormalities.

Given these repetitive behaviors, it is not surprising BDD is comorbid with other psychiatric conditions including unipolar major depression, social anxiety disorder, substance use disorders, obsessive-compulsive disorder (OCD), and eating disorders. Additionally, a series of studies showed 80% of individuals with BDD experienced suicidal ideations in their lifetime, and among those, 24–28% attempted suicide [2, 3]. The annual rate of completed suicide in patients with BDD is 0.3%, which is exceedingly higher than rates in the vast majority of other mental disorders [4]. BDD most frequently presents in adolescence but has been noted to occur across the lifespan. Those with early-onset BDD (diagnosed at age 17 and younger) were more likely to have a gradual onset, severe symptoms, and a history of physical violence due to BDD and psychiatric hospitalization [5]. Once a diagnosis of BDD is made, it generally follows a chronic course. In youth, patients are more likely to develop major functional impairment, reduced academic performance, social withdrawal, and school dropout. Into adulthood, these patients have higher rates of occupational impairment, unemployment, social dysfunction, and social isolation [6]. Recognizing and providing appropriate treatment for BDD might decrease the probability of developing subsequent conditions and suicide attempts.

Yet, studies have shown that patients with BDD do not disclose concerns about their appearance unless specifically questioned (Table 2). While the estimated point prevalence of BDD in the general population is 2-3%, ineffective physician-patient dialogue leads to underdiagnosis [7]. Two meta-analyses suggested that the psychiatric inpatient and outpatient setting prevalence was 7% and 6%, respectively [8]. Within the inpatient psychiatric setting specifically, it is essential to recognize BDD to prevent exacerbations of the primary disease itself and other comorbid conditions. Furthermore, maintenance treatment for body dysmorphia is crucial, as its estimated recovery occurs only in 20% of patients [9]. Initiating and maintaining treatment requires a high index of suspicion by the physician in order to facilitate thorough history taking, inquiring about symptoms consistent with BDD, and making a clinical diagnosis.

It is important to distinguish BDD from normal appearance concerns, which are common in the adolescent years. BDD goes undiagnosed due to multiple reasons. Patients may experience shame and embarrassment about their symptoms, or they desire nonmental health treatment such as cosmetic surgery. Patients often have a poor prognosis due to the lack of insight.

## 2. Case Report

A 40-year-old female presented to the emergency department (ED) with a 4-month history of suicidal ideation. The patient self-reported to the ED due to new onset plan to commit suicide via overdose on prescription medication. The patient reported gradually increasing feelings of tiredness, hopelessness, and self-loathing, as well as anhedonia, increased appetite, and diminished libido. Columbia-Suicide Severity Rating Scale (C-SSRS) revealed the patient to be high risk due to answering the following questions affirmatively: have you had suicidal thoughts and had some intention of acting on them, have you started to work out or worked out the details of how to kill yourself, and do you intend to carry out this plan [10]. Preparatory behaviors included buying liquor to drink in combination with prescription medication, including her father's heart medication, as well as, taking time off of work to spend with her best friend prior to her planned attempt. She had no previous aborted or interrupted suicide attempts. Per the ED physician, the patient was interactive and showed no outward signs of distress. Physical exam revealed no abnormalities. Vital signs were as follows: blood pressure 132/53 mm Hg, heart rate 84 beats/minute, respiratory rate 16 breaths/minute, oxygen saturation 98% on room air, temperature 36.6 C, and BMI 28.57 kg/m^2^. Laboratory values were all normal including comprehensive metabolic panel, complete blood count with differential, thyroid-stimulating hormone, ethanol, urine drug screen, and urinalysis.

The patient denied past inpatient or outpatient psychiatric diagnoses or treatment. Past medical history included thyromegaly and tobacco use disorder for which she was previously prescribed bupropion for smoking cessation. She admitted to using alcohol and marijuana. She regularly drank 1 glass of wine daily for the past few years; she noted an increase in consumption the couple of weeks prior to her hospital admission but did not exceed 4-5 drinks per day and did not drink to the point of intoxication. She described her marijuana use as less than monthly.

**Figure 1:**
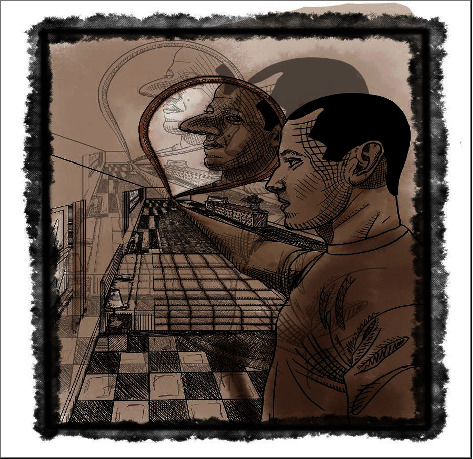
This image was painted by an artist during the patient's inpatient hospitalization to illustrate the feelings that the patient experiences.

During her inpatient hospitalization, it was revealed she had a dysfunctional relationship with her mother, which led to feelings of bodily defects in regard to body shape beginning at age 8 (Figure 1). She noted the size of her thighs and abdomen in relation to her peers as her primary concerns. She routinely engaged in mirror checking as a result. These feelings persisted into adulthood and carried with them other comorbid psychiatric conditions. She began experiencing symptoms of depression at age 15, including suicidality, lack of interest in previously enjoyed activities, little sleep, and energy. She stopped attending events where crowds would be involved because she felt judged by strangers; inability to interact in social settings led to personal and professional impairment. Shortly thereafter, she started having intrusive thoughts about the state of cleanliness of bathrooms and kitchens. She admitted to repetitively cleaning the areas for greater than an hour at a time and had to scrub surfaces an even number of times. The amount of time she spent cleaning was concerning to her family; their knowledge of her habits was another source of distress. At age 40, the symptoms of her psychiatric conditions were exacerbated and compounded with suicidal ideations. She was able to recognize and discuss her symptoms with multiple providers and was immediately willing to participate in her treatment plan.

During her inpatient admission, she was administered the Yale-Brown Obsessive-Compulsive Scale (Y-BOCS) test and received a score of 27 [11]. Of note, she reported spending more than 3 and up to 8 hours per day on compulsive behaviors with definite interference with social or occupational performance. She had a very strong drive to perform compulsive behaviors and was only able to delay with difficulty. The patient was subsequently diagnosed with body dysmorphic disorder with good insight, OCD with good insight, and mild alcohol use disorder. She began treatment with sertraline 50 mg daily but was transitioned to escitalopram oxalate 10 mg daily after she experienced gastrointestinal discomfort. She attended group therapy, received exposure and response prevention therapy, cognitive behavioral therapy interventions, and was provided psychoeducation on distress tolerance skills and behavioral activation. She identified whenever she was experiencing stress, she would focus on coping painting. She was introduced to the rubber band technique to break habitual behaviors and stop negative thoughts. Her week-long stay in the inpatient psychiatric unit did not involve any acute exacerbations, and she was discharged to home. She is continuing to receive psychiatric care on an outpatient basis including continuing escitalopram oxalate 10 mg daily, prazosin 1 mg daily ,and trazodone 100 mg nightly for sleep, as well as psychotherapy.

## 3. Discussion

Patients with body dysmorphic disorder have great potential to be misdiagnosed in clinical settings due to comorbid psychopathology masking the underlying condition. In fact, one study demonstrated that unipolar major depression was present in 75%, social anxiety disorder in 40%, substance use disorders in 30-50%, and OCD in 33% of patients with BDD [12]. Most patients with body dysmorphic disorder present with an average of 2.5 comorbid conditions [13]. Numerous studies have demonstrated the onset of BDD to precede development of other psychiatric disorders [5, 6, 8]. Patients report what is described as a streamline effect resulting from the impact of BDD on their functioning and quality of life. For example, alcohol use disorder stems from the desire to cope with the negative affect from major depressive disorder and BDD.

Even after making the diagnosis of BDD, the course of illness is further perplexed by the vast patient response to pharmacotherapy and psychotherapy. Cognitive behavioral therapy (CBT) is the first-line treatment for BDD. CBT for BDD should begin with assessment and psychoeducation to inform, explain, and assimilate biological and psychosocial factors to individualize treatment and relapse prevention plans. Collaborative empiricism is imperative for CBT effectiveness. CBT techniques that can aid in BDD treatment and maintenance are cognitive restructuring, self-monitoring, exposure and ritual prevention (E/RP), perceptual retraining, and relapse prevention [14].

E/RP consists of collaborative empiricism in developing the hierarchy of anxiety provoking situations and avoidance behaviors. Once identified, validation of feelings, and evidence to support the rationale for exposure will be given. The objectives of E/RP are to assist individuals with BDD in practicing distress tolerance skills and gaining insight [15].

Inpatient psychiatric care is used primarily for symptom stabilization in cases of mental health crisis. BDD symptoms may become severe requiring psychiatric hospitalization. Criteria for psychiatric inpatient admission may be met when an individual with BDD is not able to keep up with daily responsibilities or poses immediate risk or danger to themselves [7, 13, 14]. Brief psychological interventions and lengths of stay restrict treatment of BDD in an acute care, inpatient setting. Delivery of CBT and E/RP techniques will vary but require greater lengths in treatment than this setting can provide. Treatment during admission will consist of targeted interventions focused on achieving immediate goals and stabilization. Successful treatment and remission of BDD require patients to develop skills in identifying maladaptive thoughts and cognitive errors, restructuring cognitions and core beliefs, behavioral experiments and strategies, relapse prevention, and access to posttreatment booster sessions. Due to the complexity of BDD, patients may be initially reluctant to disclose symptoms and may present only with depressive symptoms and/or expression of suicide intent. BDD is commonly missed in these patients or can be misdiagnosed as OCD due to the similarity of BDD's and OCD's characterization of obsessions and compulsive behaviors [16]. However, if patients can be screened and diagnosed with BDD, treatment can be initiated through psychoeducation and brief interventions during the course of hospital stay. Patients will be connected with outpatient appointments to access further treatment to manage and cope with BDD symptomology [8].

## 4. Conclusions

BDD is a frequently encountered and debilitating illness that appears in an amalgam of clinical settings. This disorder can cause horrendous suffering and poor functioning in many aspects of life. It is important to recognize characteristic symptoms that prompt investigation of BDD rather than assume other conditions are responsible for the patient's current presentation. This will lay the foundation for implementing an appropriate treatment regimen targeting BDD specifically. In turn, other psychiatric conditions may be mitigated or disappear entirely.

## Figures and Tables

**Table 1:** DSM-V-TR diagnostic criteria for body dysmorphic disorder.

(a) Preoccupation with one or more perceived defects or flaws in physical appearance that are not observable or appear slight to others (b) At some point during the course of the disorder, the individual has performed repetitive behaviors (e.g., mirror checking, excessive grooming, skin picking, and reassurance seeking) or mental acts (e.g., comparing his or her appearance with that of others) in response to the appearance concerns (c) The preoccupation causes clinically significant distress or impairment in social, occupational, or other important areas of functioning (d) The appearance preoccupation is not better explained by concerns with body fat or weight in an individual whose symptoms meet diagnostic criteria for an eating disorder. Specify if (i) *With muscle dysmorphia*: the individual is preoccupied with the idea that his or her body build is too small or insufficiently muscular. This specifier is used even if the individual is preoccupied with other body areas, which is often the case Indicate degree of insight regarding body dysmorphic disorder beliefs (e.g., “I look ugly” or “I look deformed”). (ii) *With good or fair insight*: the individual recognizes that the body dysmorphic disorder beliefs are definitely or probably not true or that they may or may not be true (iii) *With poor insight*: the individual thinks that the body dysmorphic disorder beliefs are probably true (iv) *With absent insight/delusional beliefs*: the individual is completely convinced that the body dysmorphic disorder beliefs are true.

**Table 2:** Screening questions for BDD.

(1) Are you worried about your appearance in any way? Or are you unhappy with how you look? (2) If the patient replies affirmatively, proceed as you would with any other illness, asking the patient to tell you about their concerns (3) Ask if there are other body areas that they do not like (4) Next, ascertain that the patient is preoccupied with those perceived flaws by asking “how much time would you estimate that you spend each day thinking about your appearance, if you were to add up all the time you spend? Or do these thoughts preoccupy you? (5) Ask “how much distress do these concerns cause you?” after the patient replies, ask more specifically whether the concerns cause anxiety, social anxiety, depression, panic, or suicidal thinking (6) Ask about effects of the appearance preoccupation on the patient's life. After the patient replies, ask more specifically about effects on (i) Work (ii) School (iii) Social life (iv) Any other aspects (7) While BDD behaviors are not required for the diagnosis, most patients perform at least one of them (usually many), and it is important to ask about the common ones: camouflaging, comparing, mirror checking, excessive grooming, seeking reassurance, touching the body areas, clothes changing, skin picking, tanning, dieting, excessive exercise, and excessive weightlifting

## Data Availability

The data used to support the findings of this study are included within the article.
